# Caspase 3 activity in isolated fetal rat lung fibroblasts and rat periodontal ligament fibroblasts: cigarette smoke induced alterations

**DOI:** 10.1186/1617-9625-11-25

**Published:** 2013-12-06

**Authors:** Asra Ahmed, James A Thliveris, Anthony Shaw, Michael Sowa, James Gilchrist, James E Scott

**Affiliations:** 1 Departments of Oral Biology, University of Manitoba and Manitoba Institute for Child Health, Winnipeg, Canada; 2 Human Anatomy and Cell Science, University of Manitoba and Manitoba Institute for Child Health, Winnipeg, Canada; 3 The National Research Council Biodiagnostics Institute, University of Manitoba and Manitoba Institute for Child Health, Winnipeg, Canada; 4 The Biology of Breathing Group, University of Manitoba and Manitoba Institute for Child Health, Winnipeg, Canada; 5 Department of Oral Biology, Faculty of Dentistry, University of Manitoba, Winnipeg, Manitoba, Canada

**Keywords:** Cigarette smoke extract, Fetal rat lung fibroblasts, Protease, Caspase-3, Lung development, Developmental toxicity, Periodontal ligament fibroblast, Periodontitis

## Abstract

**Background:**

Cigarette smoking is the leading cause of preventable death and has been implicated in pathogenesis of pulmonary, oral and systemic diseases. Smoking during pregnancy is a risk factor for the developing fetus and may be a major cause of infant mortality. Moreover, the oral cavity, and all cells within are the first to be exposed to cigarette smoke and may be a possible source for the spread of toxins to other organs of the body. Fibroblasts in general are morphologically heterogeneous connective tissue cells with diverse functions. Apoptosis or programmed cell death is a crucial process during embryogenesis and for the maintenance of homeostasis throughout life. Deregulation of apoptosis has been implicated in abnormal lung development in the fetus and disease progression in adults. Caspases are proteases which belong to the family of cysteine aspartic acid proteases and are key components for downstream amplification of intracellular apoptotic signals. Of 14 known caspases, caspase-3 is the key executioner of apoptosis. In the present study we explored the hypothesis that cigarette smoke (CS) extract activates caspase-3 in two types of fibroblasts, both of which would be exposed directly to cigarette smoke, isolated fetal rat lung fibroblasts and adult rat periodontal ligament (PDL) fibroblasts.

**Methods:**

Isolated fetal rat lung fibroblasts and adult PDLs were used. Cells were exposed to different concentrations of CS for 60 min. Caspase-3 activity and its inhibition by Z-VAD-fmk were measured by caspase-3 fluorometric assay. The effect of CSE on cellular viability was measured using the MTT formazan assay. Caspase-3 expression was detected by western blot analysis and cellular localization of caspase-3 was determined by immunofluorescence using fluorescence microscopy.

**Results:**

It was observed in fetal rat lung fibroblast cells that CSE extract significantly (p<0.05) increased caspase-3 activity and decrease cell proliferation. However, no significant changes in activity or viability were observed in PDLs.

**Conclusions:**

This indicates CS activates caspase-3 the key regulatory point in apoptosis in fetal rat lung fibroblast cells suggesting that smoking during pregnancy may alter the developmental program of fetal lung, jeopardizing the establishment of critical cellular mechanisms necessary to expedite pulmonary maturation at birth.of critical cellular mechanisms necessary to expedite pulmonary maturation at birth.

## Background

Cigarette smoking leads to exposure to more than 4000 known chemicals many of which are carcinogenic and is the leading cause of preventable premature death in the world. There is strong evidence that smoking during pregnancy is strongly associated with growth retardation [1], low birth weight, sudden infant death syndrome (SIDS), preterm delivery and higher incidence of stillbirth [2]. Moreover, cigarette smoke absorption begins in the oral cavity and many studies have provided convincing evidence that CS exposure is a risk factor for oral pathological conditions ranging from staining of teeth, discoloration of the gingival to degradation of tooth supporting structures [3]. The oral cavity and the respiratory system are the primary regions for exposure to toxic substances in cigarette smoke. The mechanisms by which changes occur in these regions due to cigarette smoke exposure are not clearly elucidated.

The respiratory system is one of the most complex systems of the body which originates from two primary germ cell layers, the endoderm and the mesoderm [4]. Electron microscopic studies have identified that developing lungs undergo dramatic tissue growth and remodeling to achieve a mature architectural structure and apoptosis plays an important role during these stages [5]. Synchronization of cell proliferation, differentiation and apoptosis is the basis of organogenesis. Any defect in the apoptotic processes during embryogenesis may lead to developmental abnormalities and damage to cells due to metabolic stress [6]. It is well known that the lungs have to be sufficiently mature at birth in order to acquire normal gas exchange of oxygen and carbon dioxide for survival in the extra-uterine environment. The immature lung is susceptible to toxic substances in cigarette smoke primarily because of the fewer number of macrophages leading to a poor immune response [7] and also due to a decrease in surfactant quantity, which is considered as the first line of defense against pathogens [8]. Fetal growth and development is influenced by the intrauterine environment and governed by physical, environmental, genetic and hormonal factors [9]. It has been reported that maternal smoking of more than 20 cigarettes per day is associated with high risk for fetal hypoxia [10]. Previous studies noted that chronic fetal smoke exposure is a major predisposing factor for SIDS [11]. At the cellular level tobacco smoke induces apoptosis and reduces cell viability in human fetal lung fibroblast-derived cells [12]. Moreover, cigarette smoking appeared to induce apoptosis via a caspase-dependent pathway in isolated human type I cells [13].

The periodontal ligament (PDL) is a unique and dynamic soft connective tissue which is a highly cellular connective tissue with fibroblasts as the predominant cell type. The PDL forms a fibrous joint, primarily responsible for anchorage of tooth to alveolar bone. Degradation of this connection between the tooth and bone may lead to loss of attachment, increased tooth mobility eventually leading to tooth loss [14]. PDL fibroblasts are responsible for the high turn over rate, rapid remodeling capacity during injury and repair. This necessitates continuous involvement of the apoptotic process for maintenance of PDL integrity. Since the greater part of the PDL is formed by fibroblasts, exposure to toxic substances in tobacco smoke is a potential risk factor for these cells.

Caspases are proteases which belong to the family of cysteine-aspartic acid endo-peptidases. Caspases are primarily localized in the cytoplasm and are synthesized as inactive enzyme precursors or zymogens [15]. The activation of caspases leads to irreversible biochemical and morphological changes in cells. Caspases can be broadly classified into two groups; one which is thought to play a central role in apoptosis (caspases -2, -3, -6, -7, -8, -9, -10, and -12) and another group which are primarily involved in cytokine processing during inflammation (caspases -1, -4, and -5) [16] Caspase-3 has been reported to contribute mainly to the characteristic morphologic changes in apoptotic cells including membrane blebbing, chromatin condensation and DNA fragmentation (19) functional role in the initiation and execution of apoptosis. Recent studies suggest that activation of initiator caspases is by autolytic cleavage within a linker segment separating the large and small subunits due to an intrinsic proteolytic activity of the caspase zymogen (salvesen and riedl, 2008). Once activated the initiator caspases activate effector caspases [17]. The effector caspases lack the ability of selfactivation because of their small pro-domains. Activation of initiator caspases does not always result in apoptosis, due to the fact that members of anti-apoptotic family (Bcl-2) can prevent the activation of effector caspases [18]. Caspase-3 is the first of all the effector caspases to be activated for amplifying downstream apoptotic process. Caspase-3 can be activated through caspase-8 and caspase-9 by extrinsic or intrinsic signaling, respectively [19], suggesting, that the apoptotic signal from either extrinsic or intrinsic pathways converge for the activation of caspase-3. Furthermore, activation of caspase-3 is a very rapid process in the cell death process [20] which is associated with mitochondrial membrane permeabilization [21].

The aim of the present study was to examine the effects of cigarette smoke extract on fetal lung fibroblasts and adult rat PDL fibroblasts. We hypothesized that CS extract induces activation in these cells through caspase-3.

## Methods

### Preparation of cigarette smoke extract

Cigarette smoke extract (CSE) was prepared according to method designed by [22]. Unfiltered research cigarettes 2R1 from University of Kentucky, each containing 2.45 mg nicotine/cigarette [23] were used. CS was drawn from a single cigarette into a 50 ml syringe for two seconds maintaining a gap of 20 seconds between each draw with the syringe and bubbled through 50 ml of MEM at room temperature. This cycle was repeated till the end of the cigarette and then 50 ml of fresh MEM was used for the next cigarette. The resulting smoke extracted MEM was considered to be 100% CSE. It was filtered using 0.22 μm pore filters (Millipore) making it sterile and free from contaminants and stored at –80°C. Further dilutions (5%, 10% and 15%) (v/v) were made in serum free media containing antibiotics and fungizone. Before treating cells with conditioned media, pH was adjusted to 7.2. Samples of 100% CSE were analyzed by gas chromatography on a Varion 320MS TQ gas chromatograph to determine nicotine content. Final nicotine content was determined to be 30 μg/ml of medium which translates to 0.20 μM.

### Isolation and culture of fetal rat lung fibroblasts

Pregnant Sprague–Dawley rats purchased from Central Animal Services, University of Manitoba were used to isolate fetal lung fibroblasts. Rats were euthanized with an intraperitoneal injection of 1 ml Euthanyl (240 mg/ml sodium pentobarbital) on gestational day 21 (day 23 is term gestation). Fetuses were removed by hysterotomy, decapitated and placed in cold, sterile Hanks Balanced Salt Solution (HBSS, Gibco, ON Canada). Lungs were dissected from fetuses by making an incision in the mid-sternal region, and minced using a Sorval tissue chopper (Sorval Instruments, Newton, CT) in a laminar flow hood. The minced lung tissue was dissociated by incubating with trypsin-EDTA (0.05%/0.02%) in HBSS at 37°C for 45 minutes in a water-jacketed trypsinization flask which was placed on a magnetic stirrer. Minimal essential medium (MEM) (Gibco, ON Canada) containing 10% of newborn calf serum (NCS), antibiotics/antimycotic (1%) and fungizone (1%) (Gibco, ON Canada) was added to stop further enzymatic disaggregation. The dissociated cells were filtered through three layers of 150 μm Nitex gauze to remove tissue fragments and centrifuged for 10 min at 1000 rpm at 4°C. The cell pellet was re-suspended in 10 ml of MEM/NCS and cells were plated in five 75 cm^2^ tissue culture flasks in a humidified incubator (95% air/ 5% CO_2_) and allowed to adhere for 1 hour. Fibroblasts have the ability to attach faster when compared to type II cells [24]. After this period media from each flask containing non-adherent cells (including type II cells, RBC’s) was collected in an autoclaved beaker. Fresh media (10% NCS) was added to the flasks which had attached fibroblasts. Media was changed after 24 hours for the first time and then at 48 hours thereafter. The cell monolayers were cultured for 3–4 days till they reached 80% confluence and sub-cultured in a ratio of 1:3. Fibroblasts were passaged five times to obtain sufficient numbers for experimentation. Purity was determined visually by phase microscopy. By confluence no epithelial-like cells could be detected.

### Culture of rat periodontal fibroblasts

Rat PDL fibroblasts were kindly supplied by Dr. C. Lekic and had been isolated according to the method outlined by Lekic et al. [25]. The cells were thawed and placed in 25 cm^2^ flasks. Media was changed at 24 hours for the first time and every 48 hours thereafter until they reached 70-80% confluence and sub-cultured in larger flasks in the ratio of 1:3, cultured till 70-80% confluent for further use in experiments.

### Detection of caspase-3 activity in adherent cells exposed to CSE

Once the cultures reached 70-80% confluence, they were washed twice with HBSS and treated with different concentrations of CSE diluted in serum free media for 60 min. After which the cells were washed three times with HBSS to ensure complete removal of traces of CSE. As the key regulatory enzyme in activation of apoptosis [26], caspase 3 activity in cells was determined following instructions of caspase-3 fluorometric assay kit, purchased from BioVision, (MountainView, CA). The cells were lysed using the lysis buffer available with the kit (50 μl per well) and incubated on ice for ten minutes, with 5 μl of fluorogenic substrate 1 mM DEVD-AFC (caspase 3 cleaves to this substrate) in a reaction buffer (containing 10 mM DTT) in the incubator at 37°C for two hours. The enzymatic activity was monitored using a fluorescence microplate reader with 400 nm excitation and 505 nm emission filter. Caspase 3 cleaves the AFC substrate and releases a fluorogenic signal; this signal is directly proportional to the level of enzymatic activity of caspase 3 in cells. Caspase 3 activity was calculated in treated samples and compared to untreated controls.

### Effect of N-benzyloxycarbonyl-Val-Ala-Asp-fluoromethylketone (Z-VAD-fmk)

*Z-VAD-fmk,* is a broad spectrum caspase inhibitor which was used in the present study to examine the involvement of caspases in cell death due to CS exposure. The cells were incubated with 80 mM concentration of Z-VAD-fmk in serum free media at the time of exposure of cells to CSE for 60 min. After which the cells were washed and the caspase-3 activity was measured using the fluorometric assay kit, purchased from BioVision, (MountainView, CA) as described above.

### Determination of cellular viability

The cells were treated with different concentrations of CSE as described above. After incubation with different concentrations of CSE the cells were washed with HBSS three times to ensure complete removal of CSE and further incubated with MTT solution for three hours. The MTT based cell proliferation assay (Sigma Aldrich, St.Louis, MO, USA), is a calorimetric assay used to measure the ability of mitochondrial dehydrogenase of viable cells to reduce the key component, MTT or 3-[4,5-dimethylthiazol-2-yl]-2,5diphenyl tetrazolium bromide, a yellow tetrazole to insoluble purple formazan crystals. Viable cells cleave the tetrazolium ring of MTT and the yellow water soluble dye is converted to insoluble purple crystals of formazan. After three hours of incubation with MTT solution the crystals were dissolved in MTT solvent by pipetting three times in order to completely dissolve the crystals. The plates were read spectrophotometrically at an absorbance of 570 nm. The intensity of purple color in the solution results in an increase in absorbance level, indicative of the number of living cells.

### Western blot analysis

At the end of treatment with CSE, cells were washed three times with HBSS to ensure complete removal of any remnants of CSE. Cells were lysed by adding one ml of 2XRIPA buffer with protease inhibitor tablet [20 mM Tris–HCl pH 7.6, 316 mM NaCl, 2 mM EDTA, 2% triton X100, 0.2% SDS, 2% sodium deoxycholate, 1 mM PMSF, 1 mM Na_3_VO_4_, 1 protease inhibitor tablet] and stored at −80 until processing. Protein samples were quantified using Bradford protein determination method. Equal amounts of protein extracts were subjected to onto 12% sodium dodecyl sulfate-polyacrylamide pre-cast gels (BIO-RAD, Mississauga, ON), electrophoresed at 180 V and later transferred to nitrocellulose membranes. The blots were probed with primary antibody (rabbit polyclonal) purchased from Santa Cruz Biotechnology (Santa Cruz, CA, USA) and diluted (1:500) in blocking buffer overnight at 4°C. This antibody recognizes p17 fragment of an activated form of Caspase 3 (Santa Cruz, CA, USA). After three washes with TBS-T blots were incubated with goat anti rabbit IgG-HRP secondary antibody (Santa Cruz, CA, USA) at a dilution of 1: 1,000 in blocking buffer for two hours at room temperature and detected using ECL-plus (GE Healthcare, NJ, USA) and exposed on Kodak films. The densities of cleaved caspase 3 bands were quantified using Quantiscan.

### Sub-cellular localization of caspase-3 using immunofluorescence

Sub-cellular localization of caspase-3 in cells exposed to CSE was observed using immunofluorescence microcopy. Cells were plated in four well glass chamber slides and left overnight in incubator at 37°C for attachment on glass slide. The cells were exposed to 10% or 15% CSE in serum free media and left in the incubator for 60 min at 37°C. After which the cells were washed with PBS three times and fixed with cold methanol (−10°C) for 5 min followed by three washes with PBS, suction was used between each wash to completely remove the reagents. The cells were blocked in 2% BSA/1 × PBS for one hour in a humidified chamber. The primary anti-body rabbit-polyclonal IgG, which recognizes active caspase-3 was diluted (1:800 dilution) in blocking solution was added to the cells and incubated overnight at 4°C in a humidified chamber. After four washes with PBS cells were incubated with secondary anti body, FITC conjugated donkey anti-rabbit IgG which recognizes rabbit IgG by immunofluorescence staining (Santa Cruz). The secondary antibody was diluted 1:80 in 2%BSA/1xPBS and cells were incubated in humidified chamber for one hour in the dark. All steps after this was done in the dark. After four washes with PBS the cells were stained with Hoescht 33342 (1:1000) for 15 seconds. 4 × 5 min washes the slides were air-dried and mounted with coverslips using 40 μl Prolong Anti-fade Gold, the edges sealed with nail paint. The slides were observed under an inverted fluorescence microscope (B X 61 Olympus microscope) using ImagePro Software.

### Statistical analysis

Statistical differences between group means were carried out using *post hoc* Duncan’s Multiple Range Test [27]. A value of p < 0.05 was considered for statistically significant differences between the treated and untreated groups.

## Results

### Detection of caspase-3 activity in adherent cells exposed to CSE

**Figure 1:**
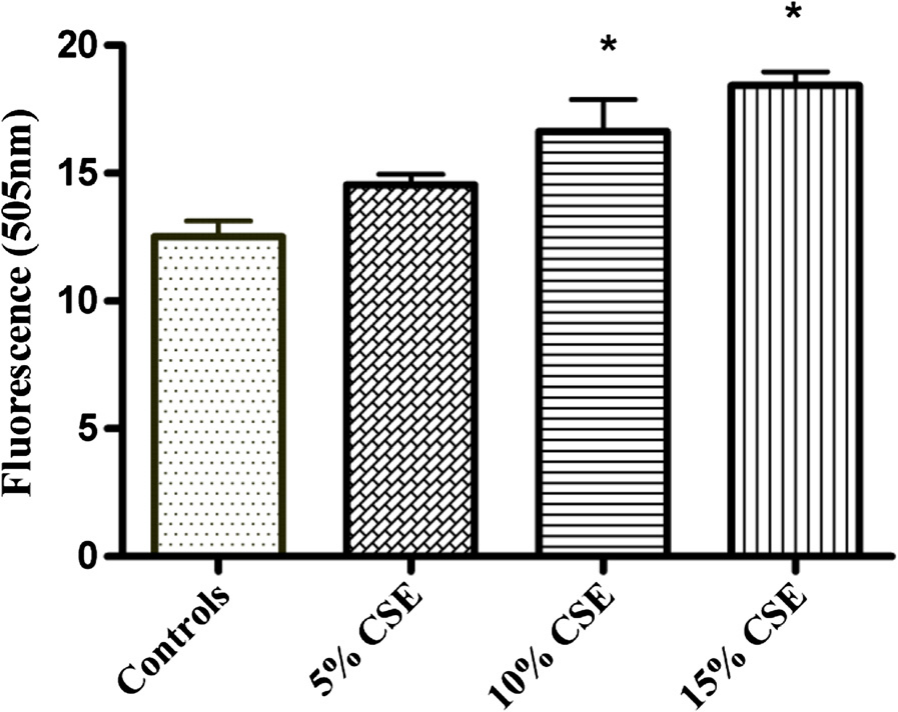
**Effect of CSE on caspase 3 activity in fetal rat lung fibroblasts.** Fluorometric assay to assess the activity of caspase-3 in fetal rat lung fibroblasts exposed to different concentrations of CSE (5%, 10% or 15%) (v/v) for 60 minutes in 37°C incubator. Cells not exposed to CSE were considered as controls. Each bar represents the mean ± SEM of three experiments of 16 samples in each. (*) indicates (p < 0.05) significantly different from the corresponding controls.

**Figure 2:**
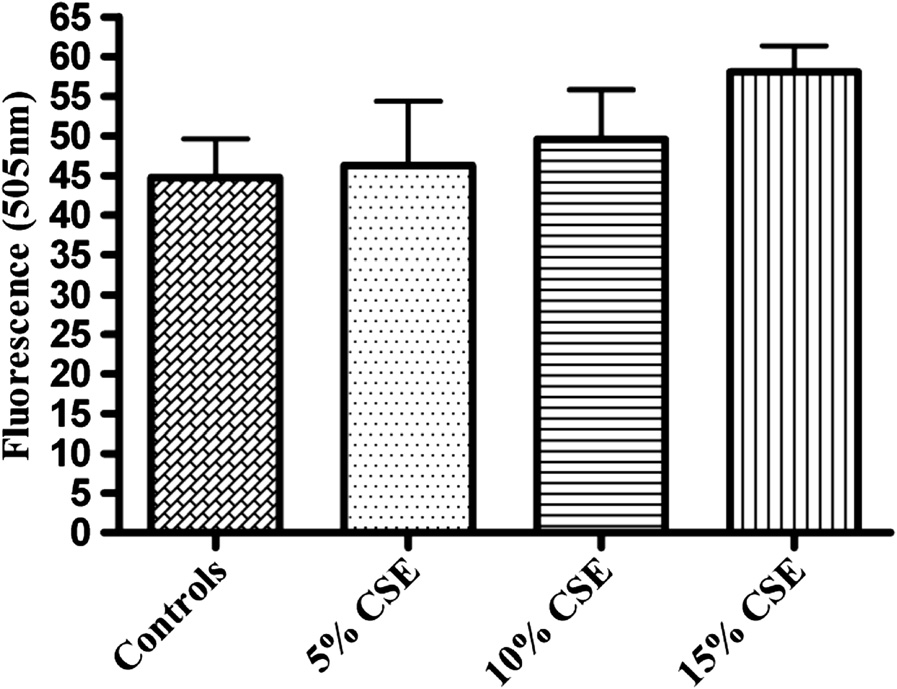
**Effect of CSE on caspase 3 activity in rat periodontal ligament fibroblasts.** Fluorometric assay to assess the activity of caspase-3 in rat periodontal ligament fibroblasts exposed to different concentrations of CSE (5%, 10% or 15%) (v/v) for 60 minutes in 37°C incubator. Cells not exposed to CSE were considered as controls. Each bar represents the mean ± SEM of three experiments of 16 samples in each. No significant differences were noted.

The main executioner caspase to be activated in the process of apoptosis is caspase-3. To further analyze Caspase-3 activity in isolated fetal rat lung fibroblasts and PDL fibroblasts exposed to CSE (5%, 10%, and 15%) (v/v) for 60 minutes a fluorometric assay kit was used. Exposure to CSE at concentrations of 10% and15% (v/v) produced significantly elevated activity (p < 0.05) of caspase-3 compared to the non-exposed cells (Figure 1). No significant differences were observed in the caspase-3 activity in cells exposed to 5% CSE when compared to the non-exposed cells. Interestingly, No significant differences were observed in the caspase-3 activity in PDL fibroblasts (Figure 2) exposed to 5%, 10% or 15% (v/v) CSE compared to the non-exposed cells.

### Effect of N-benzyloxycarbonyl-Val-Ala-Asp-fluoromethylketone (Z-VAD-fmk)

**Figure 3:**
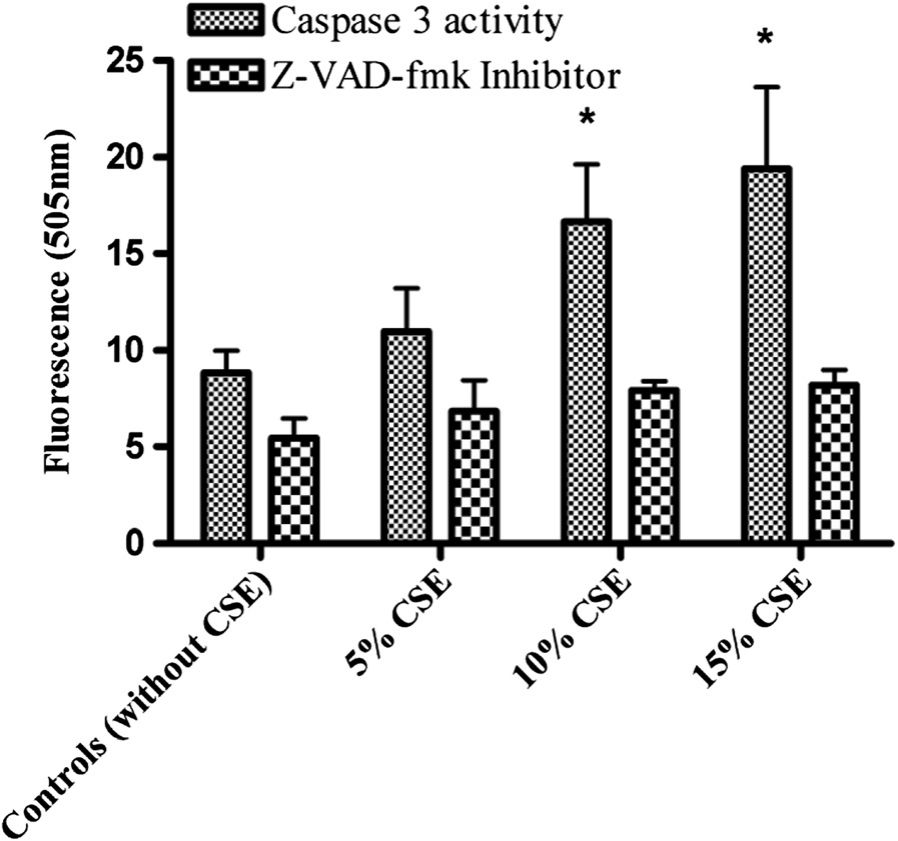
**Effect of Z-VAD-fmk and caspase 3 activity in isolated fetal rat lung fibroblast cells exposed to CSE.** Caspase 3 activity and effect of Z-VAD-fmk was measured using caspase 3 fluorometric assay in isolated fetal rat lung fibroblast cells exposed to 5%, 10% or 15% (v/v) CSE for 60 minutes. Each bar represents the mean of ± SEM of three experiments of 16 samples each. (*) indicates significantly (p < 0.05) different from the corresponding controls.

**Figure 4:**
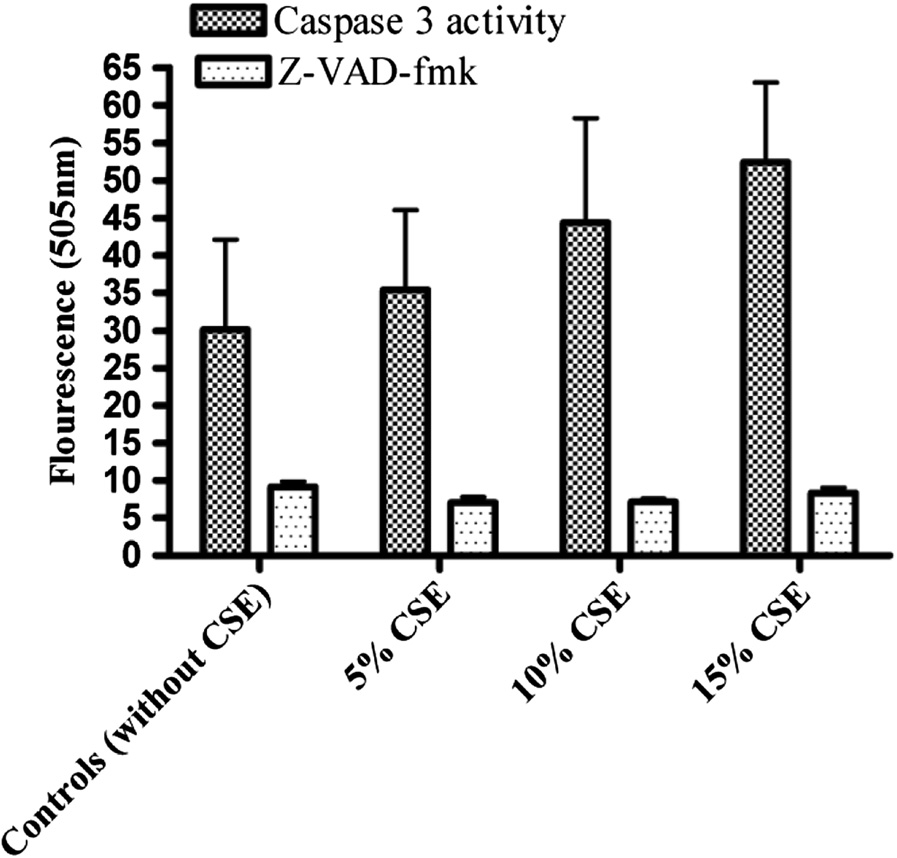
**Effect of Z-VAD-fmk and caspase 3 activity in rat PDL fibroblasts exposed to CSE.** Caspase 3 activity and effect of Z-VAD-fmk was measured using caspase 3 fluorometric assay in rat PDL fibroblast cells exposed to 5%, 10% or 15% (v/v) CSE for 60 minutes. Each bar represents the mean of ± SEM of three experiments of 16 samples each. No significant differences were noted.

For further confirmation, apoptotic cell death was evaluated after treatment with *Z-VAD-fmk*, a broad spectrum caspase inhibitor. Isolated fetal rat lung fibroblasts (Figure 3) cells and PDL fibroblasts (Figure 4) were exposed to different concentrations of CSE (5%, 10% and 15%) and Z-VAD-fmk (80 μM concentration) was added along in CSE for 60 minutes. The controls were not exposed to CSE. The caspase-3 activity in CS exposed cells with and without the Z-VAD inhibitor was measured using the fluorometric assay kit at 400 nm excitation and 505 nm emission. In all samples caspase-3 activity was inhibited by Z-VAD-fmk.

### Determination of cellular viability

**Figure 5:**
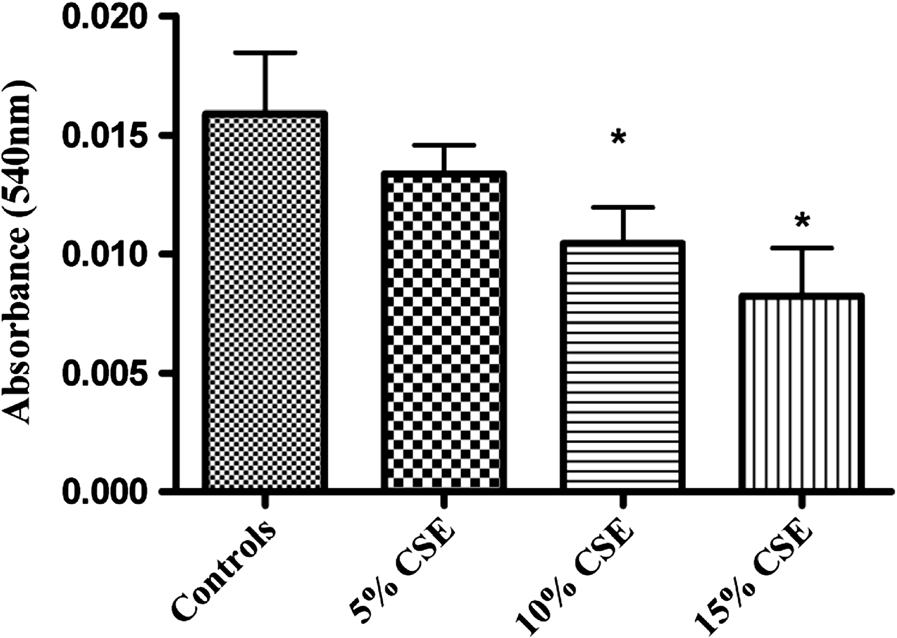
**Effect of CSE on cell viability in isolated fetal rat lung fibroblasts.** MTT activity was measured in fetal rat lung fibroblasts exposed to different concentrations of CSE (5%, 10% or 15%) (v/v) for 60 minutes. Cells not exposed to CSE were considered as controls. Level of absorbance was measured at 540 nm. Each bar represents the mean ± SEM of three experiments of 16 samples in each. (*) indicates (p < 0.05) significantly different from the corresponding controls.

**Figure 6:**
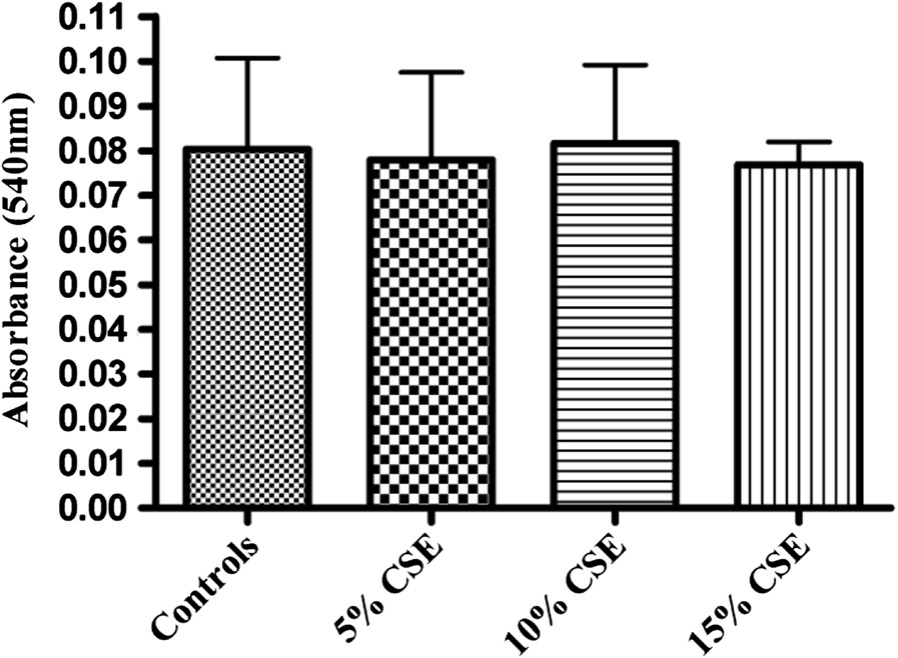
**Effect of CSE on cell viability in rat PDL fibroblasts.** MTT activity was measured in fetal rat PDL fibroblasts exposed to different concentrations of CSE (5%, 10% or 15%) (v/v) for 60 minutes. Cells not exposed to CSE were considered as controls. Level of absorbance was measured at 540 nm. Each bar represents the mean ± SEM of three experiments of 16 samples in each.

The cellular viability was measured using MTT formazan assay. The formazan assay measures cellular mitochondrial dehydrogenase activity within a cell and is based on the conversion of mitochondrial-dependent MTT, (3-(4,5-dimethylthiazol-2-yl)-2,5-diphenyltetrazolium bromide) to purple formazan crystals. There was a significant decrease in the mitochondrial activity of fetal lung fibroblasts exposed to 10% and 15% CSE (Figure 5) compared to the cells not exposed to CSE. No significant decrease or increase in the mitochondrial activity of PDL fibroblasts (Figure 6) exposed to 5%, 10% or 15% CSE was observed compared to the cells not exposed to CSE.

### Western blot analysis

**Figure 7:**
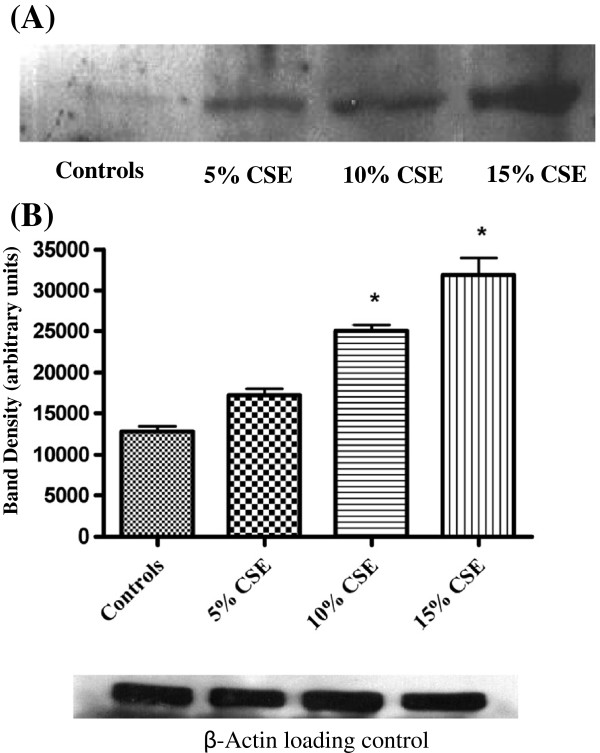
**Western blot analysis of caspase-3 from lysates of isolated fetal rat lung fibroblasts exposed to different concentrations of CSE.** Fetal rat lung fibroblasts were isolated on the 21st day of gestation and grown to 70–80% confluence in an incubator at 37°C. The cells were exposed to different concentrations of CSE (5%, 10% and 15%) (v/v) in serum free media for 60 min keeping controls which were not exposed to CSE. **(A)** Lane 1: cell lysates not exposed to CSE. Lane 2: cell lysates exposed to 5% CSE. Lane 3: Cell lysates exposed to 10% CSE. Lane 4: Cell lysates exposed to 15% CSE. **(B)** Densitometric analysis of band intensity shows each bar represents the mean ± SEM of three experiments. (*) indicates significantly different from the corresponding controls (p < 0.05). A β-actin loading control is shown beneath the plot.

**Figure 8:**
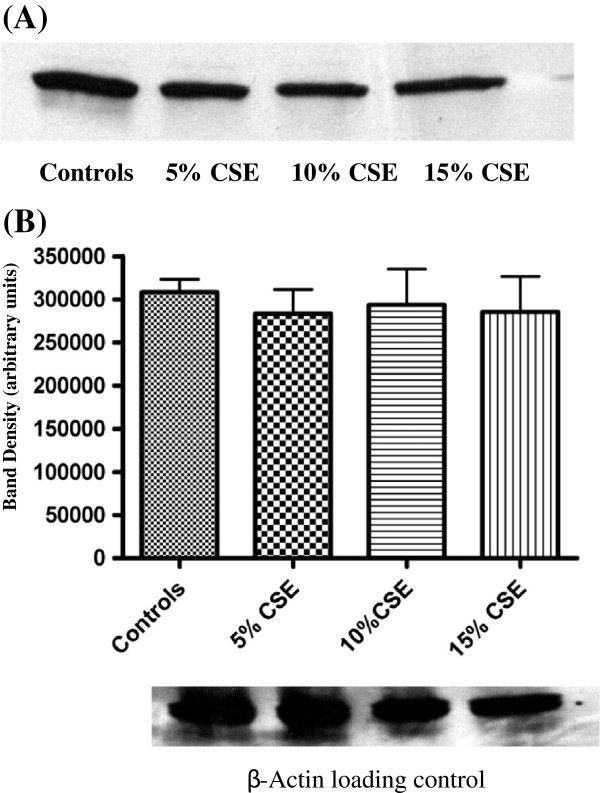
**Western blot analysis of caspase-3 from lysates of rat periodontal ligament fibroblasts exposed to different concentrations of CSE.** Rat PDL fibroblasts cultured and grown to 70–80% confluence in an incubator at 37°C. The cells were exposed to different concentrations of CSE (5%, 10% and 15%) (v/v) in serum free media for 60 min keeping controls which were not exposed to CSE. **(A)** Lane 1: cell lysates not exposed to CSE. Lane 2: cell lysates exposed to 5% CSE. Lane 3: Cell lysates exposed to 10% CSE. Lane 4: Cell lysates exposed to 15% CSE. **(B)** Densitometric analysis of band intensity shows each bar represents the mean ± SEM of three experiments. A β-actin loading control is shown beneath the plot.

The expression of caspase-3 in isolated fetal rat lung fibroblasts and adult rat PDL fibroblasts was analyzed by SDS-PAGE and Western Blotting. Lysates of cells not exposed to CSE were considered as controls. The results of fetal rat lung fibroblasts (Figure 7) and rat PDL fibroblasts (Figure 8) shows that an antibody specific for detecting active form of caspase-3 bond to the protein band with relative molecular mass of 17 kDa, which is the accepted molecular mass of active caspase-3 [28]. The densitometric analysis of caspase-3 expression in fetal rat lung fibroblasts (Figure 7) was significantly increased (p < 0.05) in the samples exposed to 10% or 15% CSE. In PDL fibroblasts (Figure 8), no significant differences were observed in the intensity of the bands exposed to CSE.

### Sub-cellular localization of caspase-3 using immunofluorescence

**Figure 9:**
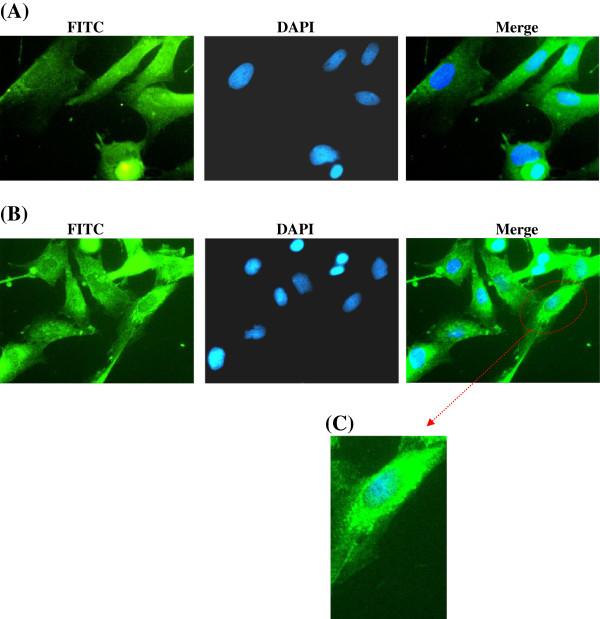
**Immunofluorescence staining for detection of caspase 3 expression in isolated fetal rat lung fibroblasts.** Isolated fetal rat lung fibroblasts were exposed to CSE (15% v/v) for three hours. Immunofluorescence was performed using caspase 3 rabbit polyclonal IGg antibody. Caspase 3 was visualized using donkey anti-rabbit IGg-FITC (green fluorescence) and counter stained with Hoescht 33342 (nuclear staining - blue). Image **(A)** shows controls not exposed to CSE. Image **(B)** shows expression of caspase 3 (green) primarily localized in the cytoplasm. Image **(C)** shows an enlarged image of a single cell.

**Figure 10:**
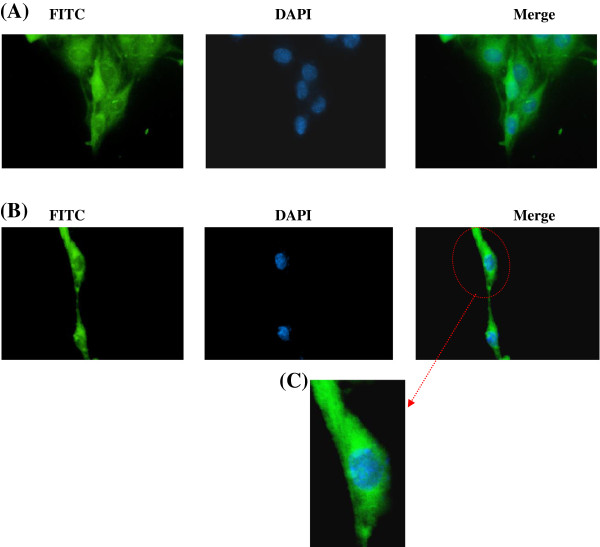
**Immunofluorescence staining for detection of caspase 3 expression in rat PDL cells.** Rat PDL cells were exposed to CSE (15% v/v) for three hours. Immunofluorescence was performed using caspase 3 rabbit polyclonal IGg antibody. Caspase 3 was visualized using donkey anti-rabbit IGg-FITC (green fluorescence) and counter stained with Hoescht 33342 (nuclear staining - blue). Image **(A)** shows controls not exposed to CSE. Image **(B)** shows expression of caspase 3 (green) primarily localized in the cytoplasm. Image **(C)** shows enlarged image of a single cell.

The sub-cellular localization of caspase-3 expression in isolated fetal rat lung fibroblasts (Figure 9) and PDL fibroblasts (Figure 10) after exposure to CSE was determined by immunofluorescence using fluorescence microscopy. The controls consisted of samples which were not exposed to CSE.

## Discussion

Among all the various types of cells present in the lungs, fibroblasts are major cells of the connective tissue and appear to be a major target for CS injury. The principal role of pulmonary fibroblasts is to maintain the integrity of alveolar structure by the synthesis, secretion, maintenance, degradation and remodeling of extracellular matrix (ECM) [29]. Furthermore, fibroblasts have been found to play an essential role in the immune mechanism of the body, through their responses to cytokines and through antigen presentation properties [30]. Fibroblasts secrete key constituents of ECM including collagen, proteoglycans and fibronectin [31] along with important biochemical mediators like growth factors and proteases. In the PDL, fibroblasts are the dominant cell type found in the periodontium. PDL fibroblasts have a major role in maintenance of the structural integrity of PDL during wound healing by synthesis of extracellular matrix. Fibroblasts construct a connective tissue lattice which firmly anchors the tooth to the alveolar bone. Previous molecular *in vitro* studies in human PDL fibroblasts suggest that nicotine was detected on the root surface of a periodontally involved tooth [3]. Salivary nicotine and cotinine levels are widely used as an indicator for total nicotine intake in the body [32]. At the cellular level, nicotine along with other toxic substances in tobacco smoke inhibit attachment of periodontal ligament fibroblasts [33]. Furthermore, the presence of chronic periodontal conditions in a regular smoker may be a potential risk factor for spread of infection leading to the development of systemic diseases [34]. Injury to the fibroblast may lead to a slower cellular turnover rate and alters the capability of repair.

In the present study we demonstrated that increasing concentrations of cigarette smoke extract (10% or 15%) induced activation of caspase-3, diminished cellular viability and cell proliferation in fetal rat lung fibroblasts in a dose-dependent manner. In contrast, adult rat PDL fibroblasts differed remarkably in their response to CSE compared to the fetal lung cells. Our animal model for the study consisted of randomly bred timed-pregnant Sprague Dawley rats. It has been clearly documented that smoking during the third trimester of pregnancy is a potential risk factor for a considerable retardation of fetal growth [35]. Additionally, isolation of lung fibroblasts during the late fetal period may facilitate the activation of caspase-3 in the late fetal period by exposure to CS. The two cell types used in the present study allowed us to compare the effects of CS on fetal fibroblasts and adult oral periodontal fibroblasts, two cell populations which are among the first to be exposed to inhaled or circulating nicotine. Indeed cells within the oral cavity often are directly exposed to cigarette by-products and have been shown to respond to nicotine treatment [36]. Fibroblasts from different regions in the body may be similar in morphology, but these cells behave differently as they are heterogeneous in nature. Morphologically, as observed through phase-contrast microscopy, the fetal lung fibroblasts and PDL fibroblasts appeared to be similar, both cells showed typical fibroblast-like appearance of being spindle shaped, elongated with a centrally located nucleus. Nonetheless, the proliferation rates of these cells differed, the fetal lung fibroblasts having a faster rate of proliferation compared to the adult PDL fibroblasts. The observations of the present study suggest that the two cell types used in this study differ from each other *in vitro*. Furthermore, studies have shown that fibroblasts of the periodontium show phenotypic differences from the gingival fibroblasts [37]. It is speculated that the difference in the periodontal and gingival fibroblasts could be due the difference in the doubling times of these two cells [37] and the difference in the origin of the cells [38]. Similarly, we speculated that the difference in behavior of fetal lung fibroblasts and PDL fibroblasts when treated with CSE *in vitro* could be due to the difference in the origin and doubling time of these two cells. PDL fibroblasts originate from the ecto-mesenchyme of the investing layer of the dental follicle and the dental papilla [39], whereas lung fibroblasts are mesenchymal in origin. Indeed the differences observed herein may relate to susceptibility to the nicotine concentrations in the CSE as Alpar et al. [40] observed that periodontal fibroblasts were only influenced by nicotine concentrations above 7.8 mM. Such a high nicotine level would most likely be toxic as determination of the levels used in the present study place undiluted CSE at 0.20 μM.

Apoptosis or programmed cell death is a normal and essential biological process for the development, maintenance of homeostasis and host defense [41]. The highly regulated process of apoptosis can be triggered by extra-cellular or intra-cellular signals resulting in cell death with the absence of inflammation. Moreover, apoptosis plays an important role during lung development and during postnatal adaptation of lung after birth for proper gas exchange [42]. Considerable research in the past supports a role of apoptosis in remodeling of lung tissue after lung injury [43]. The deregulation of apoptosis may lead to development of lung disease. Increased apoptosis in the epithelial lung cells may result in inadequate re-epithelialisation [44]. PDL fibroblasts in contrast require an ongoing process of apoptosis which is important for the maintenance of periodontal health. In response to apoptotic stimuli the inactive groups of intracellular caspases become activated and the process of cell death is carried out through proteolytic cleavage. Although caspase-3 is the central caspase in the caspase cascade that mediates the execution of apoptotic process of cell death [45], little is known about the ability of CSE to activate caspase-3 in fetal lung cells and PDL fibroblasts.

Our present *in vitro* study suggests that exposure of fetal rat lung fibroblasts to CS may trigger activation of caspase-3 leading to cell death which consequently alter cellular viability and proliferation in a dose dependent manner. We subsequently found that exposure of rat PDL fibroblasts to CS did not show a significant change in the activation of caspase-3. The activation of this enzyme in particular may result in changes in extracellular matrix secretion, remodeling and repair of injured tissues. Although the present *in vitro* study gives an insight of the changes in cellular functions, clearly further studies are required to elucidate the initiating factors inducing caspase-3 activation in smoke exposed tissues. Furthermore the complex nature of whole cigarette smoke makes it difficult to attribute the present observations to any particular component. Indeed the nonuniform nature of cigarette smoking as well as the variability of dose exposure and interpretion of this in terms of the more controlled environment of an *in vitro* study remain problematic. The identification of the critical role of caspases has been beneficial in understanding the ways by which this enzyme contributes during tissue injury and repair. Our results suggest that modulation of caspase-3 may have therapeutic potential in the prevention of lung related diseases due to cigarette smoke exposure.

## Competing interest

The authors declare that they have no competing interests.

## Authors’ contribution

AA carried out the experimental work and drafted the manuscript. JAT, JG and JES edited and revised the manuscript and supervised the work of AA. All authors read and approved the final manuscript.
